# Pharmacological Treatment of Heart Failure: Recent Advances

**DOI:** 10.2174/011573403X270178231228061314

**Published:** 2024-01-26

**Authors:** Jonathan C.H. Chan, Areeb Siddiqui

**Affiliations:** 1 Department of Medicine, Faculty of Medicine and Dentistry, University of Alberta, Edmonton, Canada;; 2 Faculty of Pharmacy and Pharmaceutical Sciences, University of Alberta, Edmonton, Canada;; 3 College of Medicine and Veterinary Medicine, Edinburgh Medical School, Edinburgh, Scotland

**Keywords:** Heart failure, SGLT2i, iron carboxymaltose, finerenone, omecamtiv mecarbil, vericiguat

## Abstract

**Background:**

Heart failure is a clinical condition with high mortality and morbidity that occurs when the heart is unable to pump enough blood to meet the metabolic demands of the body. The pharmacological management of heart failure has been revolutionized over the past decade with novel treatments.

**Objective:**

The aim of the review is to highlight the recent pharmacological advances in the management of heart failure.

**Results:**

Sodium-glucose cotransporter-2 inhibitor (SGLT2i), iron carboxymaltose, finerenone, omecamtiv mecarbil, and vericiguat have been shown to reduce hospitalization for heart failure. However, only SGLT2i, vericiguat, and omecamtiv mecarbil have been shown to reduce cardiovascular death. Finerenone has been shown to reduce cardiovascular events and renal adverse outcomes in patients with diabetes and kidney disease. Currently, only SGLT2i has been studied in patients beyond the heart failure with reduced ejection fraction population.

**Conclusion:**

The current quadruple therapy in the treatment of heart failure has demonstrated a reduction in the hospitalization of patients and a decrease in mortality associated with the condition. Individualized heart failure therapy research have shown some benefit in select heart failure patients. Further research on novel therapies will help improve heart failure patient outcomes.

## 1 INTRODUCTION

Chronic heart failure is a complex clinical condition of abnormal heart function with impaired ventricular filling or ejection of blood. Common signs and symptoms include shortness of breath, lower extremity edema, increased jugular venous pressure, and breathlessness [1].

The current pillars of quadruple heart failure therapy include angiotensin receptor—neprilysin inhibitors (ARNI), beta blocker, sodium-glucose cotransporter-2 inhibitors (SGLT2i), and mineralocorticoid receptor antagonists (MRA), all of which reduce mortality. In recent years, there have been rapid advances in heart failure therapy.

This review will focus on recent and ongoing pharmacotherapy advancements in heart failure including SGLT2i, iron carboxymaltose, finerenone, omecamtiv mecarbil, and vericiguat.

## 2 SODIUM-GLUCOSE COTRANSPORTER-2 INHIBITOR IN HEART FAILURE

SGLT2i, originally a class of antihyperglycemic medication, has been quickly adopted as foundational disease-modifying pharmacotherapy in heart failure. There continue to be advances in SGLT2i in regards to indication of SGLT2i across the spectrum of left ventricular ejection fractions.

### 2.1 Sodium-Glucose Cotransporter-2 Inhibitor in Heart Failure Mechanism of Action

The antihyperglycemic action of SGLT2i is *via* the reduction of glucose reabsorption in the renal proximal tubule [2].

Multiple mechanisms of action have been proposed for its cardiovascular effects. Namely, reduced inflammation, prevention of cardiac remodeling, and improved cardiac energetics.

### 2.2 Sodium-Glucose Cotransporter-2 Inhibitor in Heart Failure Trials

The DAPA-HF trial randomly assigned 4744 heart failure with reduced fraction (HFrEF) patients to either dapagliflozin or placebo (Table **1**) [3]. In patients who received dapagliflozin, there was a reduced occurrence of worsening heart failure or cardiovascular mortality (HR, 0.74; 95% CI 0.65–0.85; *P*<0.001). Additionally, both hospitalization for heart failure (HHF) (HR, 0.70; 95% CI 0.59–0.83) and cardiovascular mortality (HR, 0.82; 95% CI, 0.69–0.98) were reduced by dapagliflozin regardless of diabetic status.

The EMPEROR-Reduced trial randomly assigned 3730 HFrEF patients to either empagliflozin or placebo [4]. Cardiovascular death or HHF was reduced by empagliflozin (HR, 0.75; 95% CI, 0.65-0.86; *P*<0.001).

The SOLOIST-WHF trial randomized 1222 patients with type 2 diabetes and HHF to either sotagliflozin or placebo [5]. Sotagliflozin reduced cardiovascular death and HHF (HR, 0.67; 95% CI, 0.52-0.85; *P*<0.001). Of note, sotagliflozin is both a sodium-glucose cotransporter 1 and 2 inhibitor.

The EMPEROR-Preserved trial randomized 5988 patients with heart failure with preserved ejection fraction (HFpEF) with empagliflozin or placebo [6]. Empagliflozin reduced HHF or cardiovascular death (HR, 0.79; 95% CI 0.69–0.90; *P*<0.001). This benefit was seen regardless of diabetic status.

The EMPULSE trial randomized 530 patients with acute de novo or decompensated chronic heart failure, regardless of left ventricular ejection fraction, to empagliflozin or placebo [7]. The main outcome was a combination of several factors: death, occurrences of heart failure, time until the first heart failure event, or a significant change of 5 points or more in the Kansas City Cardiomyopathy Questionnaire Total Symptom Score. Patients who received empagliflozin experienced clinical benefit (stratified win ratio of 1.36 (95% CI, 1.09–1.68; *P*=0.0054).

**Fig. (1):**
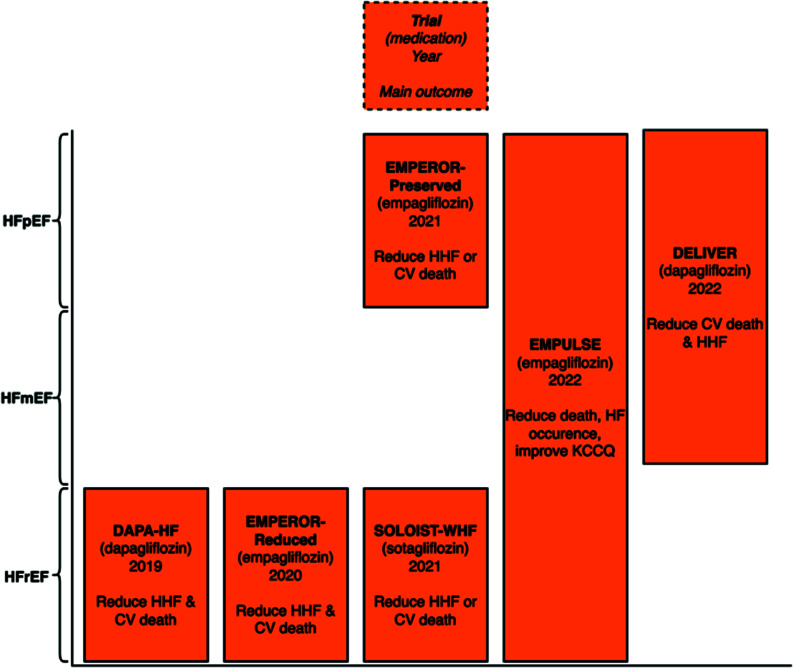
Main outcome of SGLT2i heart failure trial by left ventricular ejection fraction.

The DELIVER trial randomized inpatients and outpatients with left ventricular ejection fraction (LVEF) over 40% (or previously with HFrEF) to dapagliflozin or placebo [8]. Cardiovascular death and HHF were reduced by dapagliflozin (HR, 0.82; 95% CI, 0.73 - 0.92; *P*<0.001). Recently, a pooled meta-analysis of the DAPA-HF and DELIVER trials showed mortality and morbidity benefits across the entire LVEF spectrum (Fig. **1**) [9].

### 2.3 Sodium-Glucose Cotransporter-2 Inhibitor Safety

Overall, SGLT2i are safe medications. Based on the mechanism of action of increased glycosuria, the main adverse effect is the risk of genital mycotic infection is increased. This adverse effect is preventable through increased hygiene practices.

## 3 IRON CARBOXYMALTOSE IN HEART FAILURE

Iron carboxymaltose was originally indicated to treat iron deficiency anemia. However, emerging evidence shows benefits in patients with concomitant iron deficiency and heart failure. It is known that patients with both heart failure and iron deficiency anemia have higher rates of hospital admission [10].

### 3.1 Iron Carboxymaltose Mechanism of Action

The cause of impaired physical activity tolerance in patients with heart failure is multifactorial. Impaired oxygen usage, inadequate oxygen supply, and anemia are shown to cause impaired exercise tolerance [11]. Thus, using iron carboxymaltose to treat iron-deficiency anemia through restoring iron stores to generate more erythrocytes is a useful strategy to improve heart failure patient outcomes.

### 3.2 Iron Carboxymaltose in Heart Failure Trials

In the FAIR-HF trial, 459 patients with HFrEF and iron deficiency were randomly assigned 200 mg of iron carboxymaltose or placebo (Table **2**) [11]. Iron carboxymaltose showed improvements in the NYHA functional class (OR. 2.40; 95% CI, 1.55 - 3.71). There were also improvements in the 6-minute walk test and quality of life from iron carboxymaltose. These benefits extend to patients with or without anemia.

The CONFIRM-HF trial randomized 304 patients with HFrEF and iron deficiency to either iron carboxymaltose or placebo [12]. Iron carboxymaltose increased 6-minutes walk test times (*P*=0.002), benefited in Patient Global Assessment (*P*<0.047), and improved NYHA class (*P*=0.004). HHF was reduced by iron carboxymaltose (HR, 0.39; 95% CI 0.19–0.82; *P*=0.009). Rates of death were similar across placebo and treatment groups.

The AFFIRM-AHF trial randomized 1132 patients with either HFmrEF or HFrEF and iron deficiency to either iron carboxymaltose or placebo [13]. Iron carboxymaltose reduced HHF (RR, 0.74; 95% CI 0.58–0.94; *P*=0.013). However, iron carboxymaltose did not appear to reduce cardiovascular death.

In the IRONMAN trial, 1137 patients with HFrEF and iron deficiency were randomized to receive iron derisomaltose or usual care [14]. This trial occurred during the COVID-19 pandemic. Without sensitivity analysis censoring follow-ups due to the pandemic, HHF and cardiovascular death were not significantly reduced by iron derisomaltose (RR, 0.82; 95% CI 0.66-1.02; *P=*0.070). However, after censoring follow-ups due to the COVID-19 pandemic, a significant reduction in the primary outcome occurred in the iron derisomaltose group (RR, 0.76; 95% CI 0.58-1.00; *P*=0.047).

The HEART-FID trial consists of 3065 patients with HFrEF and iron deficiency [15]. Patients were randomized to iron carboxymaltose or placebo. The trial is still ongoing and will evaluate the outcomes of death, HHF, and change in 6-minutes walk test distance.

**Fig. (2):**
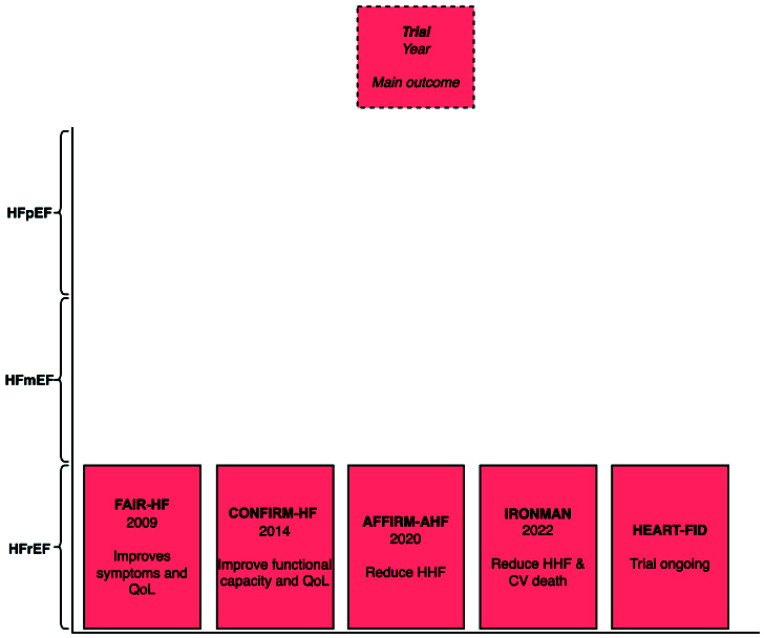
Main outcome of iron carboxymaltose heart failure trial by left ventricular ejection fraction.

Recently, iron carboxymaltose was approved by the FDA for the treatment of HF. In summary, iron carboxymaltose appears to reduce HHF, improve exercise capacity, and improve symptoms in HFrEF patients with iron deficiency (Fig. **2**). However, iron carboxymaltose was not shown to reduce cardiovascular death.

### 3.3 Iron Carboxymaltose Safety

The most common adverse effect of iron carboxymaltose is hypophosphatemia, which is caused by an increase in urinary phosphate excretion from the upregulation of the phosphaturic hormone fibroblast growth factor 23 (FGF23) [16]. To prevent iron-induced hypophosphatemia, it is important to treat underlying conditions causing blood loss as ongoing iron deficiency has shown to increase FGF23 expression.

## 4 FINERENONE IN HEART FAILURE

Finerenone is a selective nonsteroidal mineralocorticoid receptor antagonist. It has been shown to reduce cardiovascular events in patients with chronic kidney disease and type 2 diabetes, as observed in the FIDELIO-DKD trial [17]. In addition to its benefits in chronic kidney disease and type 2 diabetes, recent trials show benefits in heart failure.

### 4.1 Finerenone Mechanism of Action

As a selective nonsteroidal mineralocorticoid receptor antagonist, finerenone inhibits the binding of aldosterone and mineralocorticoid receptors. The activation of the mineralocorticoid receptor in the heart exacerbates heart failure, myocardial remodeling, and fibrosis [18]. Thus, with finerenone inhibiting the activation of the mineralocorticoid receptor, cardioprotective effects are observed.

### 4.2 Finerenone in Heart Failure Trials

The FIGARO-DKD trial randomized patients with chronic kidney disease and type 2 diabetes to receive finerenone or placebo [19]. Finerenone lowered the incidence of HHF (HR, 0.71; 95% CI, 0.56-0.90) (Table **3**).

**Fig. (3):**
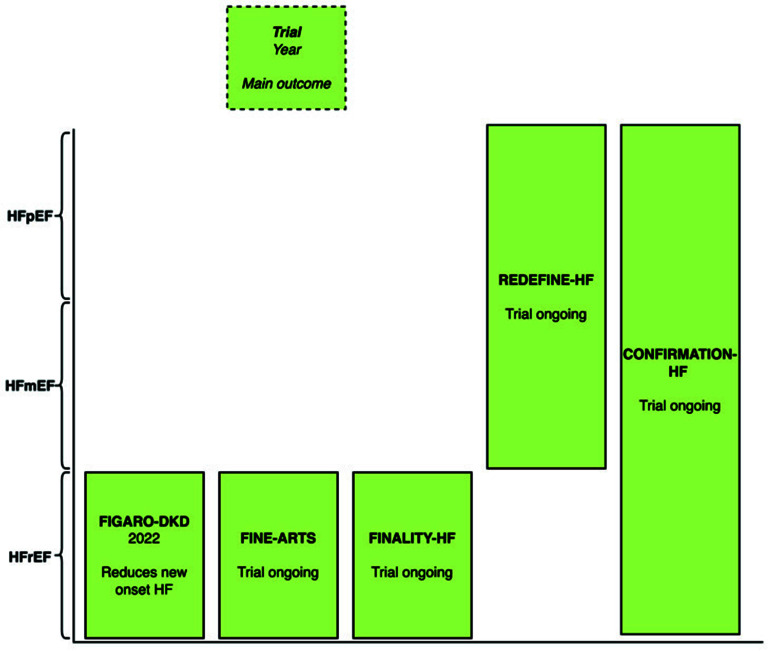
Main outcome of finerenone heart failure trial by left ventricular ejection fraction.

The FINEARTS-HF trial is ongoing and will evaluate the efficacy of finerenone in heart failure patients with a left ventricular ejection fraction greater or equal to 40% (LVEF≤40%) (Fig. **3**) [20].

Finerenone is approved by the FDA for the treatment of patients who have been hospitalized for heart failure and have CKD associated with T2D.

Only recently announced, the MOONRAKER heart failure clinical trial program includes three trials that will investigate the role of finerenone across LVEF [21].

The FINALITY-HF trial will investigate the role of finerenone in heart failure patients with a left ventricular ejection fraction of less than 40%. The REDEFINE-HF trial will evaluate finerenone in patients with left ventricular ejection fractions greater than 40%. The CONFIRMATION-HF trial will assess finerenone combined with an SGLT2i versus usual care in patients hospitalized or recently discharged with HF, regardless of their left ventricular ejection fraction.

### 4.3 Finerenone Safety

The most common adverse effect of finerenone is hyperkalemia. Routine monitoring of potassium and management can minimize such adverse effects [22].

## 5 OMECAMTIV MECARBIL IN HEART FAILURE

### 5.1 Omecamtiv Mecarbil Mechanism of Action

Omecamtiv mecarbil a cardiac myosin activator, is designed to improve cardiac performance through increasing the ability of myosin binding to actin during depolarization and also improving the efficiency of actin-independent noncontractile energy usage [23]. This helps to increase stroke volume while efficiently consuming oxygen.

### 5.2 Omecamtiv Mecarbil in Heart Failure Trials

The COSMIC-HF trial randomized 448 patients with HFrEF to either fixed-dose or pharmacokinetic-titration of omecamtiv mecarbil or placebo [24]. Patients in the pharmacokinetic-titration group had increased systolic ejection time and stroke volume, while left ventricular end-systolic and end-diastolic diameters were reduced (Table **4**).

**Fig. (4):**
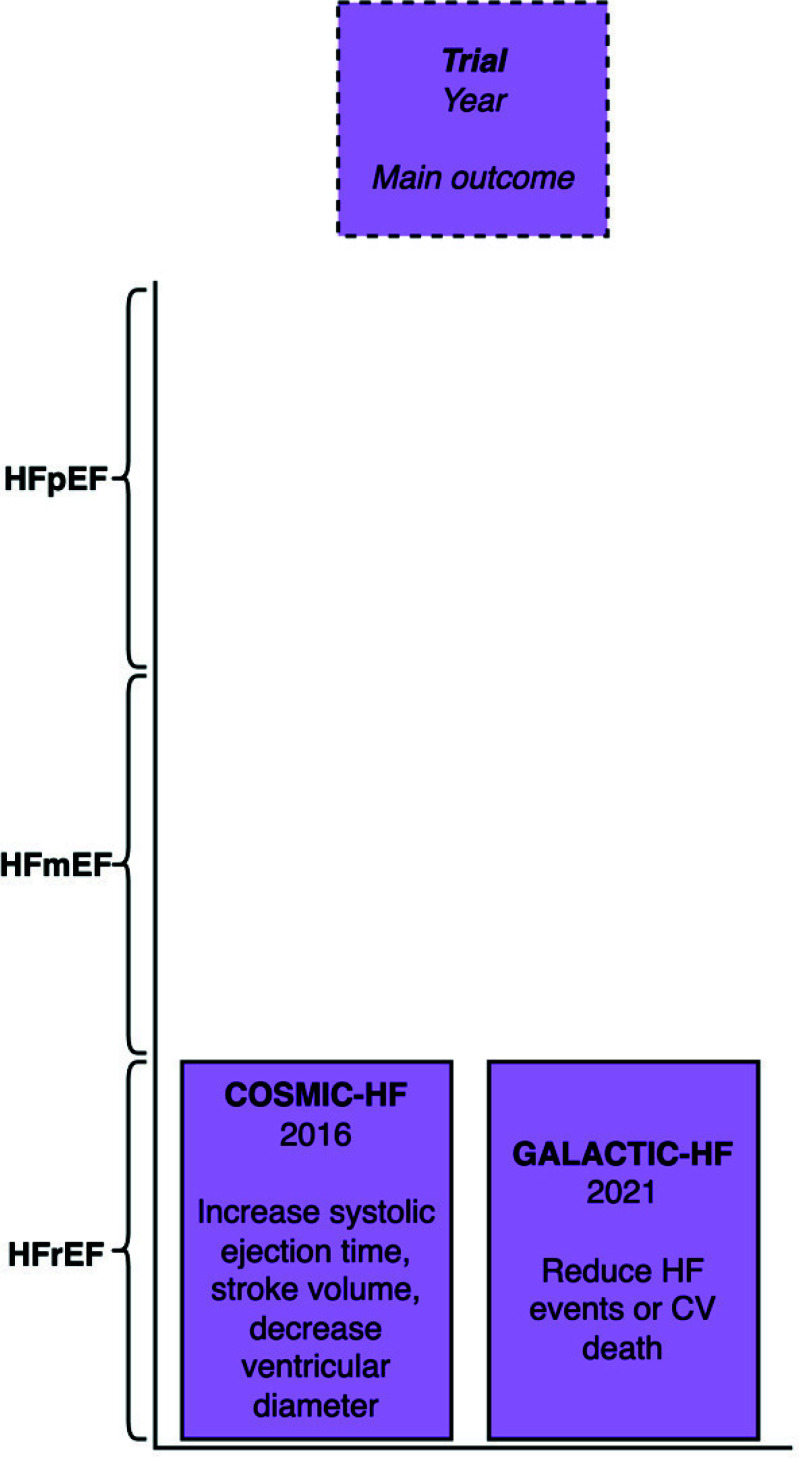
The main outcome of omecamtiv mecarbil heart failure trial by left ventricular ejection fraction.

The GALACTIC-HF trial randomized 8256 inpatients and outpatients with heart failure (EF 35% or less) to receive omecamtiv mecarbil or placebo [25]. HHF or CV death was reduced by omecamtiv (HR, 0.92; 95% CI, 0.86-0.99; *P*=0.03) (Fig. **4**).

Omecamtiv mercarbil appeared to be promising; however, recently, the FDA declined approval, citing a lack of substantial evidence and efficacy for reducing heart failure events and cardiovascular death.

### 5.3 Omecamtiv Mecarbil Safety

Omecamtiv mecarbil is a safe and well-tolerated medication. A pooled meta-analysis showed that omecamtiv mecarbil is not associated with increased death, any adverse events, hypotension, heart failure, ventricular tachycardia, dyspnea, or dizziness [26].

## 6 VERICIGUAT IN HEART FAILURE

### 6.1 Vericiguat Mechanism of Action

Vericiguat is a soluble guanylate cyclase stimulator activating the nitric oxide-soluble guanylate cyclase-cyclic guanosine monophosphate pathway, where activity is reduced in heart failure [27].

### 6.2 Vericiguat in Heart Failure Trials

The SOCRATES-REDUCED trial had 456 patients with HFrEF randomized to various doses of vericiguat or placebo [28]. Vericiguat was not statistically significant in changing N-terminal pro-B-type natriuretic peptide (NT-proBNP) levels (Table **5**).

The SOCRATES-PRESERVED trial consisted of 477 patients with HFpEF [29]. Patients were randomized to either vericiguat at various doses or placebo. Compared to placebo, vericiguat did not change NT-proBNP nor left atrial volume (LAV). Quality of life was improved by vericiguat.

In the VITALITY-HFpEF randomized trial, 789 patients with HFpEF after recent HF decompensation received either vericiguat or placebo [30]. Vericiguat was not shown to improve the physical limitation score (PLS) of the Kansas City Cardiomyopathy Questionnaire (KCCQ) in either the 10mg (95% CI, -4.6 to 3.5; *P*=0.80) or 15 mg (95% CI, −5.5 to 2.5; *P*=0.47) vericiguat groups.

The VICTORIA trial randomly assigned 5050 patients with HFrEF to vericiguat (10 mg daily) or placebo [31]. Vericiguat reduced cardiovascular death and hospitalization for heart failure (HR, 0.90; 95% CI, 0.82-0.98; *P*=0.02).

**Fig. (5):**
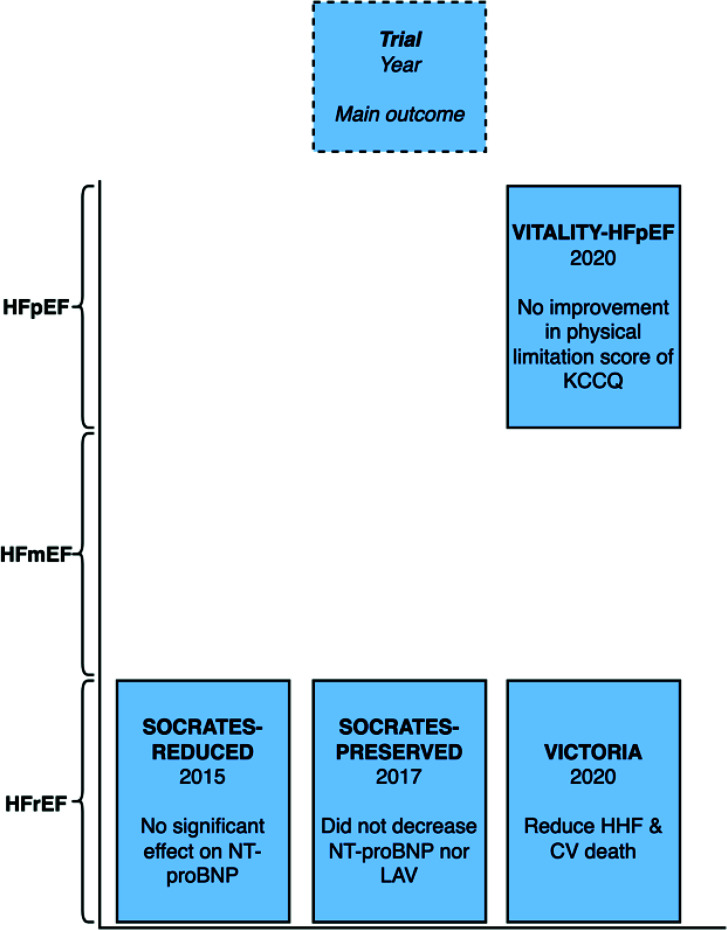
The main outcome of vericiguat mecarbil heart failure trial by left ventricular ejection fraction.

Vericiguat reduces the risk of cardiovascular death and hospitalization for heart failure. It was approved by the FDA. Vericiguat is currently indicated for patients with symptomatic heart failure with reduced ejection fraction (Fig. **5**) and where patients require hospitalization for heart failure or when outpatient intravenous diuretics are needed.

### 6.3 Vericiguat Safety

Vericiguat has been shown to be as safe as placebo in the four heart failure trials (VICTORIA, SOCRATES-PRESERVED, SOCRATES-REDUCED, and VITALITY-HFpEF) [32]. Although rare, some of the side effects associated with vericiguat include syncope, hypotension and anemia. Vericiguat is contraindicated in pregnant women due to embryo-fetal toxicity [33].

## 7 DISCUSSION

Recent advances in heart failure treatment demonstrate promising benefits. The majority of these new treatments are focused on HFrEF populations, with the exception of SGLT2i. SGLT2i were initially shown to provide benefits of reduced hospitalization for heart failure and cardiovascular death to HFrEF populations. The EMPEROR-Preserved trial and a meta-analysis of the DAPA-HF and DELIVER trials showed benefits across all ejection fractions [9].

Originally used to treat iron deficiency anemia, iron carboxymaltose demonstrated a reduction in hospitalization for heart failure and improved symptoms and was recently approved by the FDA for heart failure. The study of finerenone in heart failure patients appears preliminary. Up to this point, finerenone has only been evaluated in the FIGARO-DKD trial among patients with CKD and type 2 diabetes. The FINEARTS-HF trial is ongoing and will provide focused insights on the efficacy of finerenone in HFmEF and HFpEF patients. Vericiguat was shown to reduce cardiovascular death and hospitalization for heart failure in the VICTORIA trial. It is approved for patients with HFrEF. Omecamtiv mecarbil was shown to reduce HHF or CV death in the GALACTIC-HF trial; however, it was rejected by the FDA, citing the need to demonstrate its efficacy further.

Currently, vericiguat and SGLT2i have been adopted by heart failure guidelines [1, 34]. Vericiguat is recommended in patients with symptomatic HFrEF, in addition to standard therapy, to reduce the risk of CV mortality and hospitalizations for heart failure. SGLT2i is recommended in all heart failure patients regardless of ejection fraction.

Possible future directions include evaluating the efficacy of such therapies beyond the HFrEF population. With the FDA rejection of omecamtiv. Further studies regarding its efficacy are needed to support its approval. Future studies may evaluate the efficacy of vericiguat in HFrEF patients with high BNP levels.

## CONCLUSION

In addition to the foundation quadruple heart failure therapy (beta blockers, mineralocorticoid receptor antagonists, angiotensin receptor/neprilysin inhibitor, SGLT2i), recent advances in heart failure therapy have shown to improve patient outcomes. Most trials studying vericiguat, omecamtiv mecarbil, finerenone, and iron carboxymaltose have shown to reduce hospitalization for heart failure, with some therapies reducing cardiovascular death. While the quadruple heart failure therapy has been the standard for the treatment of patients who have heart failure with reduced ejection fraction, individualized therapy such as iron carboxymaltose has shown further benefits, including treatment in patients who have concurrent iron deficiency anemia and heart failure. These individualized therapies continue to be an area of further research.

## Figures and Tables

**Table 1:** SGLT2i heart failure trials.

**Trial (Medication)**	**Primary Outcome**	**Important Finding**
DAPA-HF (dapagliflozin 10 mg daily) [3]	Worsening HF or CV death (HR, 0.74; 95% CI, 0.65–0.85; *p<*0.001)	Dapagliflozin reduced worsening HF or CV death in both HFrEF patients with or without diabetes.
EMPEROR-Reduced (empagliflozin 10 mg daily) [4]	CV death or HHF (HR, 0.75; 95% CI, 0.65-0.86; *P<*0.001).	Empagliflozin reduced HHF and CV death in HFrEF patients, regardless of diabetic status.
SOLOIST-WHF (sotagliflozin 200 or 400 mg daily) [5]	CV death or HHF (HR, 0.67; 95% CI, 0.52-0.85; *P<*0.001).	First trial of SGLT1/2 inhibitors in hospitalized patients.
EMPEROR-Preserved (empagliflozin 10 mg daily) [6]	CV death or HHF (HR 0.79; 95% CI 0.69–0.90; *P<*0.001)	Empagliflozin reduced CV death or HHF in HFpEF patients.
EMPULSE (empagliflozin 10 mg daily) [7]	Death, HF event, time to first HF event, ≥5 change in KCCQ (score stratified win ratio, 1.36; 95% CI 1.09–1.68; *P*=0.0054)	Empagliflozin is both effective and safe in hospitalized patients.
DELIVER (dapagliflozin 10 mg daily) [8]	Worsening HF or CV death (HR, 0.82; 95% CI, 0.73-0.92; *P<*0.001)	In HFmEF or HFpEF patients, dapagliflozin reduced worsening HF or CV death.

**Abbreviations:** CV = cardiovascular; HF = heart failure; HFmEF = heart failure with mildly reduced ejection fraction; HFpEF = heart failure with preserved ejection fraction; HFrEF = heart failure with reduced ejection fraction; KCCQ = Kansas City Cardiomyopathy Questionnaire; SGLT = sodium glucose cotransporter-2

**Table 2:** Iron carboxymaltose heart failure trials.

**Trial (Medication)**	**Primary Outcome**	**Important Finding**
FAIR-HF (iron carboxymaltose 200 mg weekly) [11]	NYHA functional class (OR 2.40; 95% CI, 1.55 - 3.71)	Iron carboxymaltose improved NYHA class, 6MWT, and QOL in HFrEF and iron deficiency regardless of anemia status.
CONFIRM-HF (iron carboxymaltose 500 or 1000 mg) [12]	Improvements in: -6MWT *P*=0.002 -PGA *P*<0.047 -NYHA class improvement *P*=0.004 -HHF HR 0.39; *P*=0.009	Iron carboxymaltose improved 6MWT, PGA, NYHA class, and reduced HHF in HFrEF and iron deficiency patients.
AFFIRM-AHF (iron carboxymaltose, screening hemoglobin and body-weight based) [13]	HHF (RR, 0.74; 95% CI, 0.58–0.94; *P*=0.013)	Iron carboxymaltose reduced HHF in HFmrEF and HFrEF patients
IRONMAN (iron derisomaltose, screening hemoglobin and body-weight based) [14]	HHF (RR, 0.82; 95% CI, 0.66 - 1.02; *P*=0.070)	Iron derisomaltose is non-superior to usual care in HFrEF and iron deficiency patients.

**Abbreviations:** HFmEF = heart failure with mildly reduced ejection fraction ; HFrEF = heart failure with reduced ejection fraction; HHF = hospitalization for heart failure; NYHA = New York Heart Association; PGA = Patient Global Assessment, QOL = quality of life; 6MWT = 6-minute walk test.

**Table 3:** Finerenone trial with cardiovascular benefit.

**Trial (Medication)**	**Primary Outcome**	**Important Finding**
FIGARO-DKD (finerenone 10 or 20 mg daily) [19]	Composite of CV death, nonfatal MI, nonfatal stroke, or HHF (HR, 0.87; 95% CI, 0.76-0.98; *P*=0.03) HHF (HR, 0.71; 95% CI, 0.56-0.90)	Finerenone improved CV outcomes in patients with T2DM and stage 2 to 4 CKD

**Abbreviations:** CKD = chronic kidney disease; CV = cardiovascular; HHF = hospitalization for heart failure; MI = myocardial infarction; T2DM = type 2 diabetes.

**Table 4:** Omecamtiv mecarbil heart failure trials.

**Trial (Medication)**	**Primary Outcome**	**Important Finding**
COSMIC-HF (omecamtiv mecarbil 25 to 50 mg twice daily) [24]	Least square mean differences: -systolic ejection time 25 ms (95% CI 18-32, *P*<0.0001) -stroke volume 3.6 mL (0.5-6.7, *P*=0·0217) -left ventricular end-systolic diameter -1.8 mm (-2.9 to -0.6, *P*=0.0027) -left ventricular end-diastolic diameter -1.3 mm, (-2.3 to 0.3, *P*=0.0128)	Omecamtiv mecarbil dosed according to pharmacokinetics, improved cardiac function and decreased ventricular diameters.
Galactic-HF (omecamtiv mecarbil 25 mg, 37.5 mg, or 50 mg twice daily) [25]	HHF or CV death (HR, 0.92; 95% CI, 0.86-0.99; *P*=0.03)	Omecamtiv mercarbil reduced adverse heart failure events or CV death

**Abbreviations:** CV = cardiovascular; HHF = hospitalization for heart failure.

**Table 5:** Vericiguat mecarbil heart failure trials.

**Trial (Medication)**	**Primary Outcome**	**Important Finding**
SOCRATES-REDUCED (vericiguat 1.25, 2.5, 5, or 10 mg daily) [28]	Change in NT-proBNP level (difference of means, -0.122; 90% CI, -0.32-0.07; ratio of geometric means, 0.885, 90% CI, 0.73-1.08; *P* = 0.15)	Vericiguat did not have a statistically significant effect change in NT-proBNP level at 12 weeks
SOCRATES-PRESERVED (vericiguat 1.25, 2.5, 5, or 10 mg daily) [29]	Change in NT-proBNP level (90% CI −0.04 - 0.31) Change in LAV (90% CI −1.36 - 4.62)	In HFpEF, vericiguat was not shown to decrease NT-proBNP or LAV.
VITALITY-HFpEF (vericiguat 10 mg or 15 mg) [30]	KCCQ PLS least-squares mean difference: 15 mg group: −1.5 (95% CI, −5.5 to 2.5; *P*=0.47) 10 mg group −0.5 (95% CI, −4.6 to 3.5; *P*=0.80).	Vericiguat does not improve the physical limitation score of the KCCQ in HFpEF and recently decompensated patients.
VICTORIA (vericiguat 10 mg daily) [31]	CV death or HHF (HR, 0.90; 95% CI, 0.82 to 0.98; *P*=0.02)	CV death or HHF was lower among those who received vericiguat.

**Abbreviations:** CV = cardiovascular; HFpEF = heart failure preserved ejection fraction; KCCQ = Kansas City Cardiomyopathy Questionnaire; LAV = left atrial volume; NT-proBNP = N-terminal pro-brain natriuretic peptide; PLS = physical limitation score.

## LIST OF ABBREVIATIONS

SGLT2i: Sodium-Glucose Cotransporter-2 Inhibitor
ARNI: Angiotensin Receptor-Neprilysin Inhibitors
HHF: Hospitalization for Heart Failure
LVEF: Left Ventricular Ejection Fraction
